# Hardware Methods for Onboard Control of Fluidically Actuated Soft Robots

**DOI:** 10.3389/frobt.2021.720702

**Published:** 2021-08-17

**Authors:** Kevin McDonald, Tommaso Ranzani

**Affiliations:** ^1^ Morphable Biorobotics Laboratory, Department of Mechanical Engineering, Boston University, Boston, MA, United States; ^2^ Morphable Biorobotics Laboratory, Department of Biomedical Engineering, Materials Science and Engineering Division, Boston University, Boston, MA, United States

**Keywords:** soft robotics, soft actuator, control, smart fluids, microfluics, fluidic actuation

## Abstract

Soft robots provide significant advantages over their rigid counterparts. These compliant, dexterous devices can navigate delicate environments with ease without damage to themselves or their surroundings. With many degrees of freedom, a single soft robotic actuator can achieve configurations that would be very challenging to obtain when using a rigid linkage. Because of these qualities, soft robots are well suited for human interaction. While there are many types of soft robot actuation, the most common type is fluidic actuation, where a pressurized fluid is used to inflate the device, causing bending or some other deformation. This affords advantages with regards to size, ease of manufacturing, and power delivery, but can pose issues when it comes to controlling the robot. Any device capable of complex tasks such as navigation requires multiple actuators working together. Traditionally, these have each required their own mechanism outside of the robot to control the pressure within. Beyond the limitations on autonomy that such a benchtop controller induces, the tether of tubing connecting the robot to its controller can increase stiffness, reduce reaction speed, and hinder miniaturization. Recently, a variety of techniques have been used to integrate control hardware into soft fluidic robots. These methods are varied and draw from disciplines including microfluidics, digital logic, and material science. In this review paper, we discuss the state of the art of onboard control hardware for soft fluidic robots with an emphasis on novel valve designs, including an overview of the prevailing techniques, how they differ, and how they compare to each other. We also define metrics to guide our comparison and discussion. Since the uses for soft robots can be so varied, the control system for one robot may very likely be inappropriate for use in another. We therefore wish to give an appreciation for the breadth of options available to soft roboticists today.

## 1 Introduction

Many of the challenges facing the robotics community, such as adapting to complex and unstructured environments or performing delicate object manipulation tasks, can be addressed using soft robots (Whitesides, 2018). Soft robots embody the concept of morphological computation and physical intelligence whereby the design of the physical system, in terms of both morphology and materials, facilitates the control of the robot itself (Müller and Hoffmann, 2017; Sitti, 2021). In doing so, soft robots closely emulate the designs seen in nature and are therefore well-suited for interactions in and around biological systems (Kim et al., 2013; Pfeifer et al., 2014). They provide increased compliance and dexterity compared to traditional rigid robots, allowing them to safely navigate through delicate environments without damage to the surroundings or the robot itself (Rus and Tolley, 2015; Laschi et al., 2016). Among the most beneficial qualities of soft robots is the ability to embed many distributed degrees of freedom (DoFs), allowing for highly dexterous configurations that would be difficult to achieve with traditional robots (Laschi et al., 2016). Progress in manufacturing has led towards the development of more complex soft bodies that can integrate more advanced functionalities (Mahon et al., 2018; Ranzani et al., 2018).

Though there are many types of soft actuators, fluidic actuators are among the most common due to their ease of fabrication and ability to deliver large forces and strokes, as well as their inherent safety (Cianchetti et al., 2014; Polygerinos et al., 2017). Such actuators use pressurized fluid to inflate, causing motion. Soft fluidic actuators can be manufactured using a variety of materials and, depending on their design, are capable of various modes of actuation, including bending, extending, contracting, and twisting (Marchese et al., 2015). As such, they are well studied and have been used in a variety of applications including navigation and surgery (Shepherd et al., 2011; Moers et al., 2012; Cianchetti et al., 2014; Gorissen et al., 2017; Yin et al., 2019).

**FIGURE 1:**
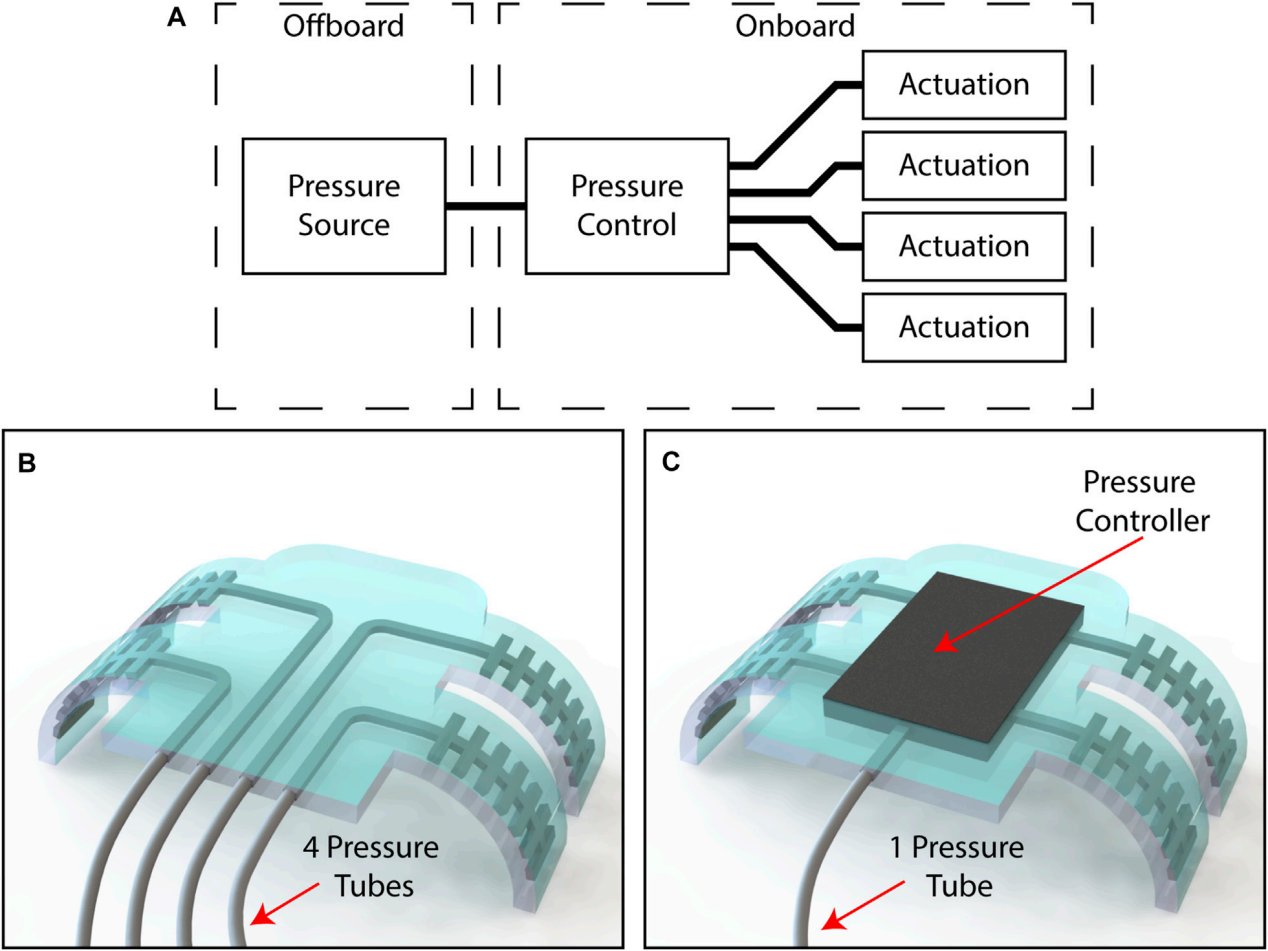
**(A)** A block diagram depicting the components that are required for a multi-DoFs soft fluidic robot **(B)** a four DoFs robot requiring four tubes to interface with an external pressure supply, and **(C)** a four DoFs robot requiring only one tube to interface with an external pressure supply due to its onboard pressure controller.

Robots capable of complex tasks generally require multiple actuators working together, and each requires some way to control the pressure (Figure 1A). While there have been efforts towards generating pressure onboard soft robots (Wehner et al., 2014; Rich et al., 2018), controlling many actuators typically requires some pressure control apparatus located externally from the robot to be connected directly to each DoF. This makes a robot controlled in such a manner inherently less autonomous due to the stiff, bulky tubes connecting the robot and the pressure supply (Figures 1B,C). The volume occupied by such tubes can be an issue where the dimensions of the working space are strictly set, such as for environments within the human body (Cianchetti et al., 2018). Pressure supply systems can also negatively impact the dynamics of the robot, leading to restrictions to the speed of operation on the order of seconds (Joshi and Paik, 2021). Fluidic soft robots are nonlinear systems and are highly sensitive to changes in the pneumatic circuit (Stanley et al., 2021).

In this review paper, we refer to control as the possibility to individually address actuators within a robot. We discuss efforts towards integrating onboard control hardware within soft fluidic robots. In many cases, such hardware is synonymous with “valves”, but we have deliberately chosen to use a broader framing so as not to exclude several relevant examples which are not valves in the traditional sense. Our use of “onboard” is meant to denote the physical location of hardware as being on or within a soft robot, and is not meant to imply any reliance on electronics or related software. We do not discuss supporting hardware such as microcontrollers or communications systems within this review, nor do we discuss software or algorithmic aspects of control. Nonetheless, we believe that the consideration of hardware components is a fundamental step toward the implementation of high-level control algorithms that will allow for compliant robots that are extensible and useful for multiple applications in the real world. Techniques from fields as varied as microfluidics and materials science have been investigated within the framework of soft robotics as a means for embedding control. These methods include traditional pneumatic and hydraulic components, microfluidic valves, macrofluidic pressure activated valves, exploiting viscous effects, and integrating smart fluids. We review efforts in embedding flow control components onboard fluidic soft robots. To do so, we present a set of criteria by which the various methods can be compared. We specifically focus on discussing fluidically-actuated soft robots.

## 2 Criteria for Comparison

A variety of strategies have been proposed to control multiple DoFs in soft fluidic robots. In this section, we introduce and define metrics that we will use to compare them, i.e., number of controllable DoFs, number of external connections, scalability, maximum pressure, bandwidth, binary vs. proportional output, use for logic, ability to reprogram, influence on robot mechanics, and manufacturing considerations.

### 2.1 Number of Controllable Degrees of Freedom

**FIGURE 2:**
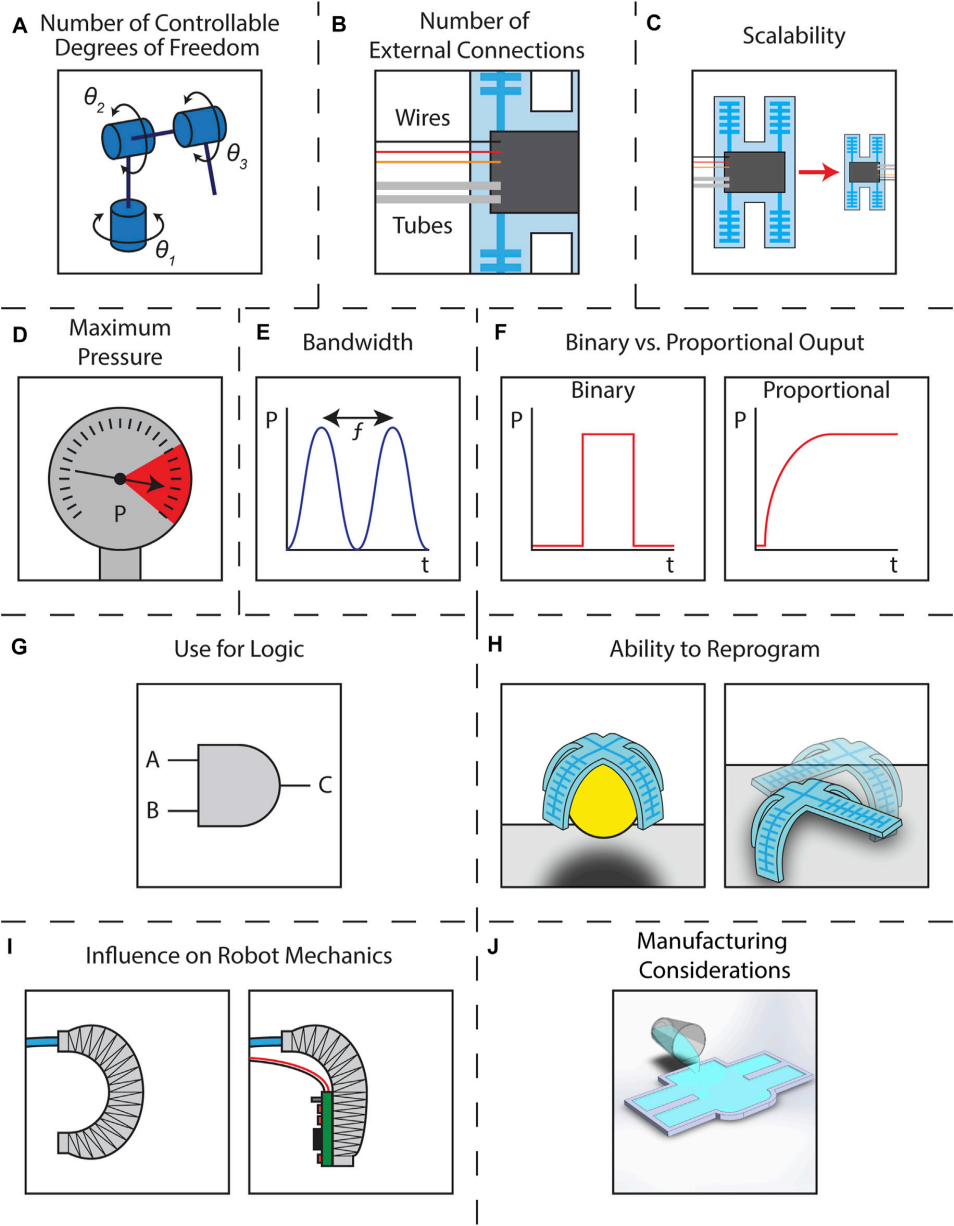
Schematics illustrating the criteria for comparison **(A)** number of controllable degrees of freedom represented by an industrial robot with three rotational DoFs **(B)** number of external connections represented by a soft robot with three wires and two fluidic tubes connected to an arbitrary controller represented by a black box **(C)** scalability represented by a soft robot manufactured at two scales with the same arbitrary controller represented by a black box **(D)** maximum pressure represented by a pressure gauge **(E)** bandwidth represented by the frequency of a pressure signal **(F)** binary vs. proportional output represented by two pressure vs. time graphs **(G)** use for logic represented by an “AND” gate **(H)** ability to reprogram represented by a robot **(left)** grasping an object and **(right)** walking across a surface **(I)** influence of robot mechanics represented by **(left)** a fiber reinforced actuator bending normally and **(right)** the same actuator having its bending inhibited by a rigid control board **(J)** manufacturing considerations represented by silicone being poured into a mold.

Of key importance for a soft robot control method is the number of DoFs it can manage. Primarily, this translates to the number of actuators the robot has (Figure 2A). The ability of a given control method to manage several actuators is among the simplest and most critical points of comparison among the various options. In our discussion of the various control methods we draw a distinction between multi-actuator systems with independently controlled DoFs and systems with coupled actuators controlled by the same pressure line, thus representing a single DoF. By doing so, we provide context for how and when the two different approaches can be useful.

### 2.2 Number of External Connections

Related to the number of DoFs controllable is the number of external connections required to control them. For a traditionally controlled fluidic soft actuator, a single line provides inflation and deflation via a connection to an offboard pressure source. Here, for every DoF there is one bi-directional external connection that provides pressurization and depressurization. The depressurization is often obtained by releasing the pressure and allowing the actuator to passively vent to atmospheric pressure. This is true for two-way valves that only have an “on” and an “off” state. Alternatively, three-way valves can route the flow to either of two external connections, one for pressurization and one for depressurization. For a robot with multiple DoFs, there are necessarily many external connections to the pressure supply. In some cases the pressure supply can be located onboard the robot, but this does not change the number of connections required for control, only where the connections lead. As in this review paper we are primarily concerned with the valves and control methods themselves and not with the issue of soft robot autonomy, we will consider any necessary connections to the valves even if they lead to systems located onboard the robot. Fluidic lines are not the only type of connection, however, since many control methods also require electrical wiring to provide power, manual control, or other operations. Such electrical connections tend to be much easier to miniaturize than fluidic ones, and pathways towards wireless communication and onboard power are much clearer for electrical systems than for their fluidic counterparts. Regardless, we will consider the external connections to a soft robot controller in much the same way one might consider the inlets of a fluidic control volume. This criterion can be visualized in Figure 2B where the fluidic control components are represented as a generic black box onboard a soft robot and the inputs are pressurized tubes and electrical lines.

### 2.3 Scalability

The scalability of onboard flow control systems relates to the size of the valves which affects the number and density of flow control components that can be integrated into a soft robot. Here we will ask: what is the smallest size the control method can be manufactured to and operate at? Valve miniaturization and density is directly related to manufacturing. Traditional valves are manufactured individually and must be combined via external connections and manifolds. By comparison, methods such as those used in microfluidics can create arrays of multiple valves simultaneously with inter-valve connections in close proximity. Additionally, compatibility of the valve manufacturing with the manufacturing of a soft robot is paramount. We will not extend “what-ifs” to hypothetical miniature versions of valves used in soft robots without evidence of their successful implementation. Additionally, some valve architectures can have issues with performance and efficiency when changing scale. We will make note when this information is available, but some of the more experimental control methods may have limited studies on this topic. As the use of soft robots increases in real-world applications with strict size constraints, such as within the human body, it is necessary that the control apparatus scales with the robot. While this is most obvious with onboard controllers, we will consider the issue of scale for all the control methods we review in this paper. For each control type, we will note the dimensional scale of both the valves themselves and the fluidic channels into which they have been incorporated, when available. This criterion is visualized in Figure 2C.

### 2.4 Maximum Pressure

Another criterion is the maximum pressure that a control method can support. Pressure is necessary to inflate fluidic soft actuators. While some robots can operate at fairly low pressure, higher pressures are typically more useful since higher pressure actuators can deliver larger forces to their environments. Some valves will break if their maximum pressure is exceeded while other control methods simply leak, but all have some maximum value. While the pressure required by an actuator is application-dependent, larger pressures and the correspondingly larger forces they can deliver are particularly useful for human-scale applications where forces on the order of newtons or more are typical. At minimum, an actuator that is expected to interact with its environment needs to be able to exert enough force to hold its own weight while completing its intended task. An untethered robot would additionally need to have actuators which could exert enough force to carry the onboard control hardware. Therefore, while not all robots require large pressures, maximum pressure is nonetheless a useful metric in choosing a valving system for a soft robot. This criterion is represented in Figure 2D by a pressure gauge reaching its maximum value.

### 2.5 Bandwidth

Soft fluidic actuators typically operate at speeds on the order of seconds or longer (Calisti et al., 2017; Yin et al., 2019), though some high speed robots can actuate in as little as 50 ms (Mosadegh et al., 2014b). To provide a metric for the bandwidth of a control method, we will consider the speed at which a valve can switch from minimum to maximum pressure. For some of the control methods, particularly those that rely on fluids other than air, the bandwidth of the system can be somewhat differentiated from the speed of the valve mechanism itself due to the dynamical interaction between the fluid and the robot. In cases of conflict, we will report the slower speed since it provides the more practical benchmark for overall performance. This criterion is represented in Figure 2E by the frequency of an oscillating pressure wave.

### 2.6 Binary Vs. Proportional Output

To see the differences between binary and proportional control for soft robots, a comparison to electronics is apt. Proportional control gives analog behavior. Like turning a potentiometer to adjust resistance, a proportional controller provides an actuator with pressures that can vary continuously between maximum and minimum values. By contrast, binary soft robot control is digital. Like one of the countless transistors switching from high to low within a computer’s processor, a soft robot controlled in a binary manner is either on or off, actuated or unactuated, inflated or deflated. While this only allows for two states predetermined by the design of the system, such control can be very quick and repeatable. For a robot that will be used to complete the same identical task over an indefinite period, this can be very useful. Binary control can also allow for complex interactions between multiple actuators, and is a prerequisite for recreating digital logic functions as described in the following subsection. It is worth noting that while methods like pulse width modulation can be used to digitally approximate an analog signal (Messina et al., 2005), we categorize such methods as proportional since we are interested primarily in the output of the soft robotic system. Binary and proportional responses are both visualized in Figure 2F as pressure vs. time curves.

### 2.7 Use for Logic

Continuing the comparison of binary soft robot controllers to digital electronics leads one naturally to consider combining such controllers to form logic systems. Like those simple transistors combining their basic switching behavior to perform complex computations, binary valves have also been used in soft robots to create a variety of more complex systems. These have been as varied as simple logic gates providing “AND”, “OR”, and “NOT” functions to combinations capable of replicating oscillators and memory. For the present, we will be highlighting the current state of logic in soft robots with an eye toward their continuously developing parity with traditional electronic systems. An “AND” gate represents this criterion in Figure 2G.

### 2.8 Ability to Reprogram

While not necessarily reliant on a logical system, the ability to reprogram a soft robot controller can be valuable and increase the versatility of the robot. While “set it and forget it” controllers are useful for repetitive tasks, a robot with reprogrammable behavior can be used for more varied tasks and in more complex environments. This programming is not strictly limited to software loaded onto some electronic microcontroller. This is certainly a possibility for some control methods, but it is also reasonable to consider devices that use some physical parameter, such as a set pressure reference or fluid viscosity, to change the robot’s behavior. Some robots allow for on-the-fly or real-time control, while others can only have their hardwired control parameters changed with direct intervention by the operator between tasks. We will note both the presence and nature of the reprogramming available with each control method discussed. This criterion is represented in Figure 2H by a robot which can be used both to grasp an object or to walk across a surface.

### 2.9 Influence on Robot Mechanics

Our penultimate criterion is the influence of the control system on the robot mechanics. The overall behavior of a soft robot is fundamentally dictated by the mechanics of its constituent materials. Thus, introducing components that affect the mechanics of the robot can affect or change its functionality. Introducing rigid control components can alter the robot mechanics and in turn reduce a robot’s speed, decrease its range of motion, or even make it unable to complete its designed task entirely. In this way, an attempt to make the robot easier to control (with regards to the pressurization of its actuators) can actually make it harder to control (with regards to its dynamics). Interfaces between soft and rigid components can additionally serve as failure points within a robot. Therefore, it is necessary to find a balance between managing the capabilities of the controller with its influence on the overall design. To provide a quantitative metric for this criterion, when available we will provide the elastic moduli of the materials comprising the valves and robots in question. This criterion is represented in Figure 2I by a soft actuator that has had its bending inhibited by a rigid control component.

### 2.10 Manufacturing Considerations

The final criterion encapsulates several considerations related to the manufacturing of each control method. While these considerations do not strictly pertain to the ultimate performance of the resulting robot, they are nonetheless important to the designers who will build said robot. We will list any equipment necessary to manufacture the valves.

We will also make explicit those methods which require specialized off-board electronics to power or control. In all cases, the original articles cited are the definitive reference for manufacturing instructions, and the summaries included here are intended only for comparative purposes such that the reader might have a sense of the complexity of the manufacturing processes. This criterion is represented in Figure 2J by silicone being poured into a mold.

## 3 Discussion of Control Methods

### 3.1 Traditional Pneumatic and Hydraulic Components in Soft Robots

**FIGURE 3:**
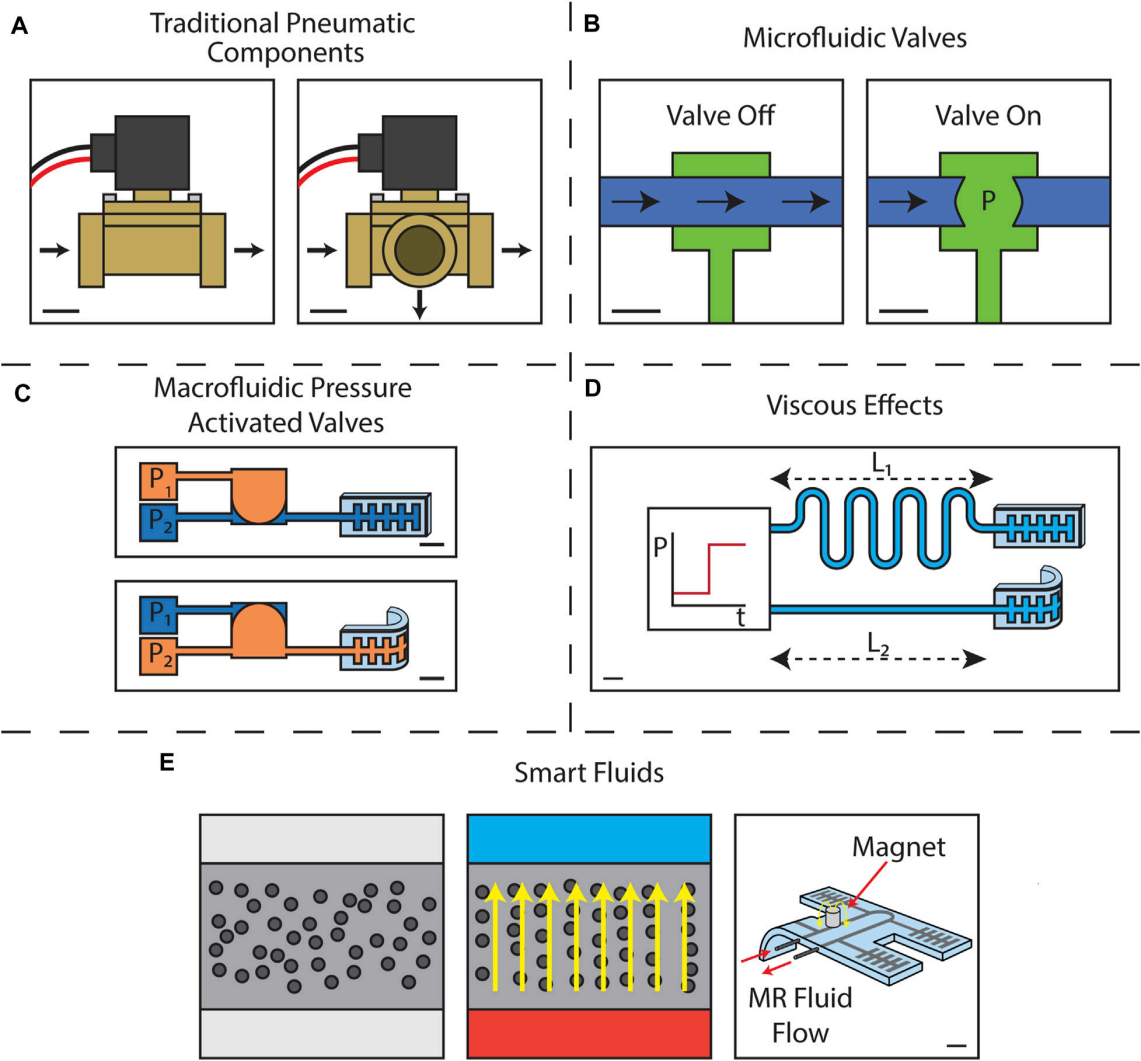
Schematics illustrating the control method types **(A)** traditional pneumatic components represented by **(left)** a two-way solenoid valve and **(right)** a three-way solenoid valve. Scale bars represent 1 cm. **(B)** microfluidic valves represented by a Quake type valve with **(left)** showing the valve off such that flow may pass and **(right)** showing the valve on such that no fluid may flow. Scale bars represent 1 mm. **(C)** macrofluidic pressure activated valves represented by an actuator-scale bistable valve with **(top)** showing *P*_1_ > *P*_2_ such that the actuator does not bend and **(bottom)** showing *P*_1_ < *P*_2_ such that the actuator bends. Scale bars represent 1 cm **(D)** viscous effects represented by two actuators with flow channels of length *L*_1_ > *L*_2_ driven by a step function pressure input such that actuator with the shorter flow channel bends first. The scale bar represent 1 cm. **(E)** smart fluids where **(left)** shows a smart fluid in the absence of an applied field **(middle)** shows the same fluid with a field applied such that the active particles align along the field lines, and **(right)** shows a soft robot filled with a continuously flowing MR fluid such that one of its actuators bends with the application of a magnetic field downstream. The scale bar in the right image represents 1 cm. The left two images are not drawn to scale.

One method for introducing control onboard a soft robot is to mount what would normally be offboard systems onto the robot itself. External control systems often have multiple valves connected to a shared pressure source and many tubes leading to the robot. Integrating the same types of valves onboard the robot can be an attractive option for reducing the number of tubes, but one must be more careful in considering the particular attributes of any given valve in accordance with the criteria set forth in Section 2. There are a variety of commercially available valves, and in this section we will discuss those which have been demonstrated for use in soft robots. A summary of these commercially available valves is presented in Table 1. We will also highlight a few custom built valves which behave similarly to commercially available devices. This category of control methods is represented in Figure 3A by two solenoid valves.

**TABLE 1:** Summary of commercially available valves used in soft robots. Detailed information is available from the manufacturers’ websites.

Manufacturer	Model	Valve type	Size [mm]	Mass [g]	Maximum pressure [kPa]	Maximum frequency [Hz]
Parker Precision Fluidics	Ten-X	2/3-way Digital Solenoid Valve	length: 32	10.7	40	200
			width: 10			
			height: 16			
Parker Precision Fluidics	X-Valve	2/3-way Miniature Pneumatic Solenoid Valve	length: 23.37	4.5	207–689	50
			width: 7.87			
			height: 12.20			
The Lee Company	120 Series	2-way Miniature Latching Solenoid Valve	length: 10.2	0.3
					34	10
			diameter: 3			
Parker Precision Fluidics	VSO MAX HP	2-way Miniature High Pressure High Flow Proportional Valve	length: 51.4	69.5	827	100
			width: 15.9			
			height: 17.4			
Parker Precision Fluidics	PND Series	Miniature Exhaust Valve	length: 25.60	7.7	41	10
			width: 13.21			
			height: 15.24			

#### 3.1.1 Examples of Traditional Pneumatic and Hydraulic Valves Used for Soft Robots

The Ten-X Digital Solenoid Valve from Parker Precision Fluidics (Parker Precision Fluidics, 2021b) has been used in multiple soft robots. This valve can support pressures up to 40 kPa and has a response time of less than 5 ms for some configurations. It is 32 mm long, 10 mm wide, 16 mm high, and has a mass of 10.7 g. The valve has barbs for 2 mm inner diameter tubing, or can be mounted in a manifold. It is manufactured out of several plastics and metals, including polybutylene terephthalate (with a Young’s modulus of about 10 GPa (AZO Materials, 2021c)) and 302 stainless steel (with a Young’s modulus of about 193 GPa (Penn Stainless Products, 2021a)). The valve is suitable for use with air and other non-reactive gases, and consumes 0.5 W. Six of these valves were used in a fully untethered crawling robot to control the application of pressure to six PneuNets actuators (Tolley et al., 2014b). The Ten-X valve was also one type of valve used to control the swimming motion of an underwater fish robot (Marchese et al., 2014).

Another off-the-shelf valve is the X-Valve Miniature Pneumatic Solenoid Valve (Parker Precision Fluidics, 2021d). This valve is available in two-way and three-way configurations. Other options are available, including a maximum pressure of 689 kPa with a 0.51 mm orifice or a maximum pressure of 207 kPa with a 0.76 mm orifice. The X-Valve offers response times as low as 20 ms in a package 7.87 mm wide by 12.20 mm high by 23.37 mm long. This valve is made of the same materials as the Ten-X valve, but has a mass of only 4.5 g. The X-Valve is also for use with non-reactive gases and consumes 0.5 W. It has been used to control the pneumatic actuators in an explosion powered jumping robot (Tolley et al., 2014a). This robot used three three-way X-Valves to deliver pressure to three pneumatic actuators which were used to position the robot prior to jumping. Two additional X-Valves controlled the mixing of the robot’s explosive fuel source. X-Valves have also been used to create an addressable pneumatic regulator for use onboard soft robots (Booth et al., 2018). At the time of writing, the X-Valve costs $39 on the manufacturer’s website.

The Lee Company offers a smaller size two-way latching 120 Series Solenoid Valve (The Lee Company, 2021). This valve is 10.2 mm long with a 3 mm diameter and has a mass of only 300 mg. The 120 Series valve supports differential pressures up to 34 kPa at a maximum bandwidth of 10 Hz. This valve provides a connection for soft tubing with an inner diameter of 1.07 mm and is itself comprised primarily of polyphenylene sulfide plastic (with a Young’s modulus of about 12 GPa (GSF Plastics Corp, 2021)) and 430 stainless steel (with a Young’s modulus of 200 GPa (Penn Stainless Products, 2021b)). The 120 Series valve is for use with air and other non-reactive gases, and consumes 1.8 W to change states. This valve has been integrated into a modular soft manipulator for use in minimally invasive surgery (Gerboni et al., 2015).

For tasks requiring a proportional output, the VSO MAX HP Miniature High Pressure High Flow Proportional Valve is available from Parker Precision Fluidics (Parker Precision Fluidics, 2021c). This valve supports proportional control of air pressures up to 827 kPa with a typical response time of 10 ms. It is 51.4 mm long by 15.9 mm wide by 17.4 mm high and has a mass of 69.5 g. The valve is available in configurations with orifices of either 2.95 mm or 3.18 mm. It is manufactured from materials including 360 HO2 Brass (with a Young’s modulus of 400 MPa (Atlanta Rod and Manufacturing, 2021)) and 300 series stainless steel (with a Young’s modulus of 193 GPa (Penn Stainless Products, 2021a)). The valve has a typical power consumption of 2.2 W. At the time of writing, the VSO Max HP Miniature High Pressure High Flow Proportional Valve costs $150 on the manufacturer’s website. It was used in the aforementioned underwater fish robot to control the inflation of its anterior pneumatic actuators (Marchese et al., 2014).

This fish robot also required an exhaust valve to be paired with the proportional valve to allow the actuators to deflate, in this case the PND Series Miniature Pneumatic Solenoid Valve from Parker Precision Fluidics (Parker Precision Fluidics, 2021a). This valve can be used to vent air pressures up to 41 kPa with a response time below 100 ms. This valve is 13.21 mm wide by 15.24 mm high by 25.60 mm long and has a mass of 7.7 g. The PND Series valve is available with orifices of 0.76 mm or 1.27 mm. Multiple materials comprise the valve, including 303 stainless steel (with a Young’s modulus 193 GPa (AZO Materials, 2021b)) and polybutylene terephthalate (with a Young’s modulus of about 10 GPa (AZO Materials, 2021c)). The PND Series exhaust valve consumes 0.5 W of power. At the time of writing, the PND Series exhaust valves costs $13 on the manufacturer’s website.

While all the above examples are available directly from valve suppliers, it is also possible to manufacture a custom valve for niche tasks. In Moers et al. (2012) a custom microhydraulic actuator was developed for use in a surgical instrument. This valve was designed to take a maximum input pressure of 800 kPa and output variable pressures from about 200 to 600 kPa at a maximum frequency of 1 Hz. It was 15 mm long and had a diameter of 4.5 mm and was designed to interface directly with McKibben actuators with a diameter of 1.5 mm. The valve was constructed from materials including aluminum (with a Young’s modulus of approximately 80 GPa (AZO Materials, 2021a)) and steel (with a Young’s modulus of approximately 193 GPa (Penn Stainless Products, 2021a)), and was designed for use with water. The valve consumed 1.2 W of power. The casing of the valve was manufactured via a turning and micromilling process.

A solenoid-like custom valve was presented by Marchese et al. (2011). This valve used an electropermanent magnet (EPM) to position a 1.5 mm ferrous ball to block air flow in a tube. EPMs, a cross between electromagnets and permanent magnets, use a coil and brief pulses of current to change the magnetic field of a soft permanent magnet. This, in conjunction with a hard permanent magnet, allows for a magnet assembly with a latching magnetic field that holds its state until another pulse is applied. Power is only consumed when sending a pulse to adjust the magnetic field, which can vary from 0 mT to a maximum dictated by the magnetic properties of the constituent permanent magnets. The magnets used to construct the valve were 6.4 mm long and 3.2 mm in diameter. They provided a peak magnetic field of 70 mT. The valve overall was 9.5 mm by 18 mm in size and had a mass of 5 g. The air tube had an inner diameter of 2.3 mm. This valve was tested with a supply pressure of 24.8 kPa and had a transition time of 0.2 s. The core of the valve was manufactured using 1,018 low carbon steel (with a Young’s modulus of 205 GPa (AZO Materials, 2012)). The exact machining process for the valve is not specified.

#### 3.1.2 Discussion of Criteria

If commercially available rigid valves are used, every DoF requires one valve to control its pressure. In many cases, pressurization and depressurization occur through the same valve, but at times it is necessary to introduce a secondary exhaust valve as with the underwater fish robot to allow the actuator to vent to atmosphere (Marchese et al., 2014). The total number of DoFs controllable within one robot is primarily limited both by cost and space constraints but is also restricted by the number of output pins available on one’s chosen control board. The robot discussed above with the largest number of DoFs had six independent actuators (Tolley et al., 2014b).

Every pneumatic or hydraulic valve has a connection to a pressure source, or atmospheric pressure in the case of exhaust valves. The pressure source can be either onboard the robot or located externally, but the external connection is typically shared between all the valves. In addition, each valve has several electrical connections including at least a ground wire and a wire to carry either a voltage or current control signal. Ground can be shared among multiple valves, but the control wires are independent. These wires pose little issue for autonomous devices with onboard control boards, but even devices with tethers can reduce the number of wire connections outside the robot by introducing electronics onboard which allow for the valves to be individually addressed (Booth et al., 2018).

Pneumatic and hydraulic valves are available at a variety of scales. However, the smallest valves tend to have the lowest performance with regards to bandwidth and maximum pressure. The smallest valve detailed above was the Series 120 valve from The Lee Company with a diameter of 3 mm and length of 10.2 mm. This valve accepted tubing with an inner diameter of 1.07 mm. The other valves had orifices which varied in size from 0.51 to 3.18 mm. In terms of area, the valves therefore had cross sections from 8 to 470 times the size of flow channels they accommodated.

Traditional valves can vary greatly with regards to the maximum pressures they can support. The valves discussed above had maximum pressures which ranged from 34 kPa for the Lee 120 series two-way latching solenoid valve to 827 kPa for the Parker VSO MAX HP Miniature High Pressure High Flow Proportional valve. Increased size is the primary trade-off for achieving higher pressures.

For soft robots, traditional valves are not a limiting factor with regards to bandwidth. As stated in Section 2.5, soft robots often operate at speeds on the order of seconds, so even the slowest pneumatic valve is likely to be faster than the robot it controls. However, solenoids with faster operating frequencies are better suited for proportional control via pulse width modulation. Faster valves can provide smoother control of the pressure when modulated in this way (Situm et al., 2007). Of the example valves with listed bandwidth information, the fastest had a response time of less than 5 ms. The slowest was the custom valve developed by Moers et al. (2012) with a maximum frequency of 1 Hz.

Traditional valves are available for both binary and proportional operation. Proportional valves tend to be more expensive, larger, and heavier. With the addition of pressure sensors, it can be possible to replicate the proportional behavior using smaller, lighter binary solenoids (Booth et al., 2018).

The use of traditional pneumatic and hydraulic valves necessitates the inclusion of a control board either offboard or integrated into the robot. As such, the valves themselves are not used to implement logic functions.

By virtue of their reliance on electronic control, traditional pneumatic and hydraulic valves are straightforward to reprogram. Electrical stimuli can be adjusted to change timing or, in the case of systems capable of proportional output, actuator pressure. Traditional valves are well suited to autonomous and untethered robots (Tolley et al., 2014b; Tolley et al., 2014a; Marchese et al., 2014) as well as robots that are controlled directly via wires (Moers et al., 2012; Booth et al., 2018) or via wireless communication (Gerboni et al., 2015).

Traditional valves can have a significant impact on soft robot mechanics. These valves contain metallic components with elastic moduli as large as 200 GPa in the case of stainless steel. By comparison, Dragon Skin 20, a common material for manufacturing soft fluidic actuators, has an elastic modulus of only 338 kPa (Smooth-On, 2021b). This drastic, six-order of magnitude difference in stiffness can introduce challenges to the inclusion of traditional valves in soft robots.

Since traditional valves are available off-the-shelf, one does not require any special manufacturing equipment to incorporate them into a robot. However, one should take care to verify the manufacturer’s requirements for power delivery and any necessary drive electronics.

### 3.2 Microfluidic Valves in Soft Robots

Many of the techniques used to develop new control methods for soft robots have been explicitly interdisciplinary, drawing inspiration from many diverse fields of study. The field of microfluidics in particular has been one of the largest influences on developing new types of soft robotics valves. In microfluidic devices, onboard flow control is achieved through pressure-activated soft valves known as Quake valves (Unger et al., 2000). Manufacturing of soft valves is done through soft lithographic processes, which consist of molding layers of elastomer cast using micromachined molds which are then bonded together chemically or via plasma bonding, creating micron-scale channels (Xia and Whitesides, 1998; Studer et al., 2004). Arranging the channels perpendicularly in separate layers allows the flow in one channel to be occluded by the pressurization of an adjacent channel. Typical flow channels and control channels have widths of approximately 100 and 250 μm, respectively, with 10 µm thicknesses (Bartlett and Wood, 2016). Typical pressure to close such a valve range from 70 to 140 kPa (Unger et al., 2000; Bartlett and Wood, 2016). These valves were demonstrated to be suitable for recreating digital logic gates within microfluidic chips, including “AND” gates, “OR” gates, inverters, and a binary decoder (Weaver et al., 2010). A basic Quake valve requires one dedicated pressurized control line for every fluid flow line, but when used in combination to produce the aforementioned logic gates it is possible to use fewer pressurized control lines. In the years since the introduction of Quake valves, the ability for microfluidic systems to replicate the computational abilities of digital electronics has continued to improve with advancements to the modeling of microfluidic logic systems, more advanced functions including oscillators and memory, and the creation of low energy bistable electrofluidic valves (Ainla et al., 2017; Woodhouse and Dunkel, 2017; Zhang et al., 2017). Dielectric elastomers and piezo actuators have been integrated into microfluidic valves to create electrically controlled devices with fewer dedicated pressure lines (Maffli et al., 2013; Tanaka, 2013; Mosadegh et al., 2014a). This narrowing gap between microfluidic and electrical logic makes the use of soft microfluidic valves an appealing choice for introducing control into soft robots where fluid is already used as the primary means of actuation. Microfluidic control methods are represented in Figure 3B by a Quake valve in its “off” and “on” states.

The Octobot is one such example of microfluidic valves used in the context of a soft robot (Wehner et al., 2016). This fully autonomous, untethered device used the decomposition of hydrogen peroxide into water and oxygen gas to pressurize its limbs. Integrated microfluidic circuitry was used to create valves and an oscillator which were used to control the alternating inflation of its limbs. The robot possessed eight total actuators, grouped into two sets of four. Due to the coupling of the limbs, this robot had two independent DoFs. The microfluidic circuitry allowed the robot to maintain its own timing and operate in a completely untethered manner so long as the fuel reservoirs remained filled. These fuel reservoirs served as the only external connections to the valving which controlled each DoF, one connection per valve. The valves themselves had maximum dimensions below 1 mm and the traces were on the order of 100 μm, a difference in magnitude of no more than 10 times. The Octobot had an operating pressure of 50 kPa, and can alternate the inflation of its DoFs at most 5.5 times per minute (a frequency of 0.0917 Hz). The valves in the Octobot were explicitly binary, and demonstrated logic via their autonomous oscillation. However, the robot could not be reprogrammed to achieve alternate behaviors. The Octobot was manufactured using a combination of molding, soft lithography, and 3D printing to build a fully soft device. The fluid-control component of the robot was manufactured using a soft lithographic process. The soft controllers were manufactured using Sylgard 184 PDMS. The robot body was in some areas manufactured from a 1:1 mix of Sylgard 184 and SE 1700 and in other areas using Ecoflex 00–30. Therefore, the valves were stiffer than some areas of the robot, but much of the robot was comprised of the same material as the valves. The soft lithographic processes used to manufacture the Octobot required several specialized tools. The molds for the soft controller were fabricated using photoresist, a process which required a spin coater, oven, UV curing apparatus, and developer. Layers of PDMS without features were also fabricated using a spincoater. A plasma system was used to bond the PDMS layers together. The inks used to 3D print the fluidic channels were created using various chemistry tools, and were made in an inert environment. They were mixed using a planetary mixer, and required a custom 3D printer to deposit. Molds for the Octobot body were fabricated out of acetal using a CNC machine. The silicones used for the body required a planetary mixer, vacuum degassing chamber, and oven. Extensive details on the fabrication of the Octobot are available in Wehner et al. (2016).

In a more recent example, microfluidic valves were used to create a soft robot capable of walking and grasping (Mahon et al., 2019). This robot used 11 fluidic switches to control its six actuators. These actuators were grouped into sets of three, for a total of two DoFs controlled. The robot used three pneumatic input lines: a vacuum line to power its actuators, a clock line for timing, and a control input line to set the robot’s state. Changing the input from low to high changed the robot from a grasping state with all actuator engaged to a walking state. In the walking state, the actuators inflated in an alternating manner at a rate set by the clock. These three external connections were shared among the 11 microfluidic switches. The robot used 1 mm channels, and the switch features were at most a few millimeters in diameter. The actuators in this robot were vacuum actuators, so the microfluidic circuitry operated between atmospheric pressure and vacuum pressure. Bandwidth information was not provided, but the operation frequency of the robot was set by external clock connection. The individual switches were binary in nature, but were used in combination for the logic that enabled the creation of a two-state machine with oscillation to control its gait in the walk state. The inclusion of the input line connection into the robot allowed it to be reprogrammed on-the-fly between its two available states. This robot used a thin silicone membrane between acrylic sheets to manufacture the microfluidic circuitry. The robot legs were also constructed from acrylic. Therefore, while the acrylic in the valves did make this robot stiff, it did not negatively impact the overall robot which was itself rigid with the exception of its soft actuators. The acrylic fluidic circuit itself was fabricated using a CNC machine. The silicone required a mixer and an oven to cure. The actuator molds were manufactured using a 3D printer, and the rigid components were made using a laser cutter.

Microfluidic valves can also be used to create a demultiplexer wherein *n* control inputs are capable of controlling 2^*n*^ outputs via a system of microfluidic switches based on Quake valves (Bartlett et al., 2020). In the specific device constructed in (Bartlett et al., 2020), four control inputs plus one primary pressure input were used to control 16 outputs. The demultiplexer was then used to demonstrate the simultaneous control of five tri-chambered soft pneumatic actuators (with one output of the demultiplexer left unused). This constituted 16 DoFs with only five external connections. Both flow channels and control channels at the point of the valves were designed to be 1,000 µm wide and 250 µm high. The membrane between the two channels varied between 54 and 112 μm, but was set to 67 µm in the demonstration device. The paper and its supplementary information provides details on the influence of the valve scale on its performance. Notably, it was determined that the membrane thickness had a larger effect on valve performance than channel width, with the pressure differential required to close the valve ranging from between 10 and 20 kPa for the thinnest membranes to as much as approximately 55 kPa for the thickest membrane. Due to the decoupling of valve performance and channel width, it was possible to manufacture many valves at high density. A pressure difference of 15 kPa or 30 kPa between the two channels was sufficient to close the valve in the demonstration device. The tri-chambered soft pneumatic actuators had a working pressure of 90 kPa with the control pressure set to 120 kPa. The valves in this paper, being very closely based on Quake valves, were binary in nature and were used together to form the demultiplexer’s logic. The demultiplexer was explicitly reprogrammable via its control inputs, allowing the bending states of the attached soft actuators to be set directly. The membranes in the demultiplexer were manufactured using MED4-4220 elastomer (with a Young’s modulus of 4.55 MPa (Avantor Sciences, 2021)). The substrate for the valves was manufactured using Sylgard 184 (Dow, 2021). The actuators themselves were manufactured using Elastosil M 4601 (Wacker, 2021). The valves therefore introduced stiffness to the overall system. The valve molds were fabricated using a combination of soft lithography and 3D printing. The lithographic process required a spin coater and UV curing system. These molds were additionally silanized in a dessicator to inhibit adhesion with the silicone. The 3D printed molds required a 3D printer and oven. The silicone molding process required a mixer, spincoater, vacuum degassing chamber, and oven. Silicone layers were bonded using oxygen plasma.

### 3.3 Macrofluidic Pressure Activated Valves

A number of pressure driven valve systems exist that, while at times similar in principle to Quake valves and the subsequent microfluidic systems discussed in Section 3.2, rely on very different manufacturing techniques. These differences and the valves’ explicit origins in the field of soft robotics are discussed in this Section. Such valves are represented in Figure 3C by an actuator-scale bistable valve.

One such example shows a bistable soft valve that uses differential pressures to control airflow through two tubes (Rothemund et al., 2018). Differently from a Quake valve, pressure did not need to be continuously applied to activate the valve. A pressure signal was used to switch the state of a bistable membrane between two stable configurations. A tube passed through each of the chambers on either side of the membrane. The pressure of each chamber could either be controlled directly or set at atmospheric pressure. Hysteretic behavior allowed the soft membrane in the center of the valve to latch in either two states, such that one tube was always kinked to prevent flow. Changing the pressure difference between the two control chambers allowed the soft membrane to snap to its opposite position, in turn changing the state of the flow through the two tubes. While the valve possesses two flow channels, these were primarily coupled together to control the state of a single DoF. The pressures in both the two flow channels and the two chambers could be controlled independently, but they could alternatively be left open to atmosphere. The valve was integrated into both a 1-DoF gripper that could autonomously grip objects and a 1-DoF earthworm-like robot that could locomote using a constant-pressure air supply. The gripper used three tubing connections since the top flow channel vented to atmosphere. The earthworm-like robot only required one tube since one chamber and one flow channel could vent to the atmosphere. The valve was 30 mm in length with a 27 mm diameter. The internal membrane had a thickness of 3 mm. The tubing connected to the valve varied from an inner diameter of 0.79–2.5 mm. The critical control pressure to switch the state of the valve was approximately 10 kPa, though the valve could withstand pressures as high as 80 kPa on the actuators before failure. The valve was shown to be capable of oscillating between states at a frequency of at most 2 Hz. This valve offered binary output between the two supply pressures, one of which was typically set to atmospheric pressure. In later works, multiples of the same valve architecture were connected to create various logical functions. Individual valves were configured as “NOT”, “AND”, and “OR” gates and combined to create latches, shift registers, and a human-robot interface to control the actuation state of a soft gripper (Preston et al., 2019b). Each logic gate had a response time of approximately 0.5  s, which when used in combination resulted in system response times on the order of seconds. A soft ring oscillator was also created which could inflate a series of actuators in a cyclic manner at a frequency of approximately 1 Hz using a single constant-pressure input 17 kPa in magnitude (Preston et al., 2019a). The valve was modified with a thinner bistable membrane to create non-volatile memory which could recall its state even after a power rest (Nemitz et al., 2020). Depending on the specific configuration, this allowed for the connected robots to have their states controlled in real time. In all cases, the valves were manufactured from silicone elastomers, including Dragon Skin 10 NV (with a Young’s modulus of 186 kPa (Smooth-On, 2021a)). The molds were manufactured using 3D printers. The silicones were mixed manually and degassed in a vacuum chamber. An oven was used during some curing steps.

A different approach is presented by Ikuta et al. (2012). Here, millimeter scale band-pass valves which opened in predetermined ranges of pressures were integrated into a catheter robot. Each band-pass valve consisted of a low-pass valve and high-pass valve connected together such that the whole valve was only open when both of the constituent sub-valves were open. This valve was used to selectively inflate two soft bellows actuators by controlling the pressure at a shared input. Check valves allowed the actuators to deflate when the input pressure was removed. To more precisely control the bending of multiple actuators simultaneously, the valves were slightly modified to respond to pulses of pressure rather than static inputs. This control method was able to control two DoFs with a single bidirectional external pressure connection. The valves were 10 mm long and 3 mm in diameter with a 1.8 mm control tube, a 1.6 times size difference. The positive pressure pulses used to control bending had an amplitude of 400 kPa and a pulse duration of 90 ms. The negative pressure pulses had an amplitude of −80 kPa. The operation of the robot, consisting of multiple pulses to orient the actuators, required approximately 40  s. In this way the valves which were themselves binary were used to deliver proportional pressures to the actuators by exploiting the time dependent response of the system. The valves were not used to create logic gates, but were themselves used as filters. This allowed for the bending of the two actuators to be controlled independently via the pressure pulse drive. The bellows actuators were manufactured from silicone. Saline was chosen as the working fluid due to biocompatibility. The valves themselves were manufactured from a UV curable resin using a hybrid microstereolithography technique developed by the authors, and as such is done using custom hardware (Ikuta et al., 1999; Ikuta et al., 2006). The authors note that even with the inclusions of small rigid components in the valves, their catheter is much less stiff than commercially available catheters manufactured entirely from stiff polymers.

In Partridge and Conn (2020), a valve is introduced that allows for rapid inflation of soft actuators. A one way valve was placed between a pressure reservoir and a soft robot consisting of two coupled PneuNets actuators and an integrated pin in the middle. When force was applied to the center of the robot, the pin was pushed through the valve, allowing both actuators to simultaneously fill with the pressurized air. This allowed the valves to control one DoF with one external connection. The pressure reservoir had an internal height of 88 mm and radius of 22.5 mm. The connected tubing had a 6 mm diameter, and the valve aperture width varied from 6 to 11 mm. The pin was 2 mm in diameter with a 30 mm length. The maximum pressure demonstrated in the work was 120 kPa. The valve worked across a range of air pressures, though the force required to open the valve was not independent of the air pressure or the soft actuator’s thickness. The vent time additionally scaled with the valve width, with the 11 mm valve venting in 0.532  s and the 6 mm valve venting in 1.298  s. The valve was binary, and was not used for logic. It also could not be reprogrammed to offer additional operation modes. The valve and soft robot were elastomeric, while the pin and pressure reservoir were rigid. Specifically, the reservoir was made of polyvinyl chloride, which has an elastic modulus of approximately 3 GPa. As the reservoir and valve were together much larger than the soft actuators, the resulting system was primarily rigid. The pin was manufactured using a 3D printer. The other components of the valve were commercially available.

A magnetic pressure-activated valve is presented by Miyaki and Tsukagoshi (2020). This valve used the balance between magnetic forces and pneumatic pressure to create a self-excited vibration from a static pressure input. Three magnets were placed around two tubes, one fixed magnet on either side and one moving magnet in the middle. The moving magnet would begin in proximity to one tube and its corresponding outer magnet, but the application of a pressure input would force the inner magnet toward the other side. Once the inner magnet moved, it uncovered a hole which would allow the recently blocked side to vent pressure. This process continued with the magnet oscillating between the two tubes until the pressure was removed. Multiples of this valve were combined to form a system that could sequentially inflate three DoFs with only one external pressure connection. The valve was 80 mm by 50 mm by 100 mm in size and had a mass of 5.1 g. At a supply pressure of 80 kPa the valve operated at 0.6 Hz. The valve was binary, and multiple valves were used in combination to build the oscillatory system. The system could not be reprogrammed except insofar as the input pressure changed the rate of operation. The magnets and the valve casing were made from rigid materials, the latter being 3D printed using acrylonitrile butadiene styrene resin. The 3D printer was the only specialized equipment necessary to produce this valve.

### 3.4 Controlling Soft Robots Via Viscous Effects

Soft robots can also be controlled directly via their constituent materials. It is possible to use the working fluid both to provide actuation and as a means to program the motion of a robot’s DoFs. This is accomplished by exploiting the material properties of the fluid, especially its viscosity. Doing so can introduce control with minimal impact on the robot’s mechanics, since all the control structures are already inherent to the actuation method. An actuator controlled via viscous effects is depicted in Figure 3D.

In an article from Vasios et al. (2019), the viscous dynamics of a pneumatic system are exploited to sequentially inflate as many as four actuators using a single pressure input. These actuators were connected in series using tubes. The radii of these tubes was carefully adjusted such that pressure would propagate through the system in a known manner. Due to the viscosity of the air and resulting frictional losses in the connecting tubes, the pressure varied across the actuators at any given point in time. The thickness of each actuator’s inflating membrane was similarly adjusted such that each actuator would inflate to their intended curvatures despite doing so at different pressures. The Navier-Stokes equations were integrated over the volume of the tube, nondimensionalized, and expressed in terms of volumetric flows to create a system of coupled differential equations which could be solved to determine the change in volume of any given actuator as a function of time. The relationship between volume and curvature was separately determined. This was used to determine the precise magnitude and duration of the input pressure pulse that would result in the desired target response. In this way, it was possible to apply different pressure inputs to the same robot to achieve different output configurations. Two different actuation behaviors were demonstrated, and the robot was shown to be capable of locomotion. The robot’s four DoFs could be inflated in a set order and at specific times, but could not be controlled in a fully independent manner. Only one external pressure connection was necessary. This control method required no valving or additional hardware besides the 0.38 mm inner diameter tubes connecting the actuators. The robot was operated at a maximum pressure of 102.7  kPa, but the mathematical model did not restrict this value and could be used to optimize a device with different performance. The time response was also captured by the model, but the actuators demonstrated in the paper inflated in 2.5  s. The control method allowed for proportional control of the actuators, and did not provide any logic functions. However, the input pressure pulse could be adjusted in magnitude and duration to reprogram the actuators to provide different responses. The robot was constructed from elastomer, with rigid plastic connectors between the tubes for ease of reconfiguration. Since the control method did not require any additional components beside the robot’s constituent parts, it did not have any negative impact on overall mechanics. The molds for the actuators were manufactured using a 3D printer. The silicone itself was degassed in a vacuum chamber.

Air is not the only fluid whose viscous effects can be exploited to control a soft robot. Silicone oil and glycerol were each used in a paper by Matia et al. (2017). Here, the dynamics of a slender beam actuator with asymmetrically distributed channels filled with viscous fluid were solved. Specifically, a modified Euler-Bernoulli equation presented by Matia and Gat (2015) was used to predict the deflection of the beam actuator while the Stokes equation and conservation of mass were used to model the incompressible creeping Newtonian flow of the working fluid. These were solved together to relate the fluid pressure to the bending of the beam. This allowed the deformation mode of the actuator to be precisely predicted and controlled by varying the inlet pressure signal. The dynamics predicted the beam would be able to exhibit simple bending, oscillatory bending, standing wave, and moving wave bending profiles. A cantilevered beam was fabricated to demonstrate the deformation modes resulting from the application of a step pressure and oscillating pressure. Only a single actuator was demonstrated, but the bending was controlled at every point along its length using a single external connection. The channels within the actuator had a diameter of 4 mm, and the actuator itself was 200 mm long by 90 mm wide by 12 mm high. The model is usable across dimensional scales, however consideration must be made to the viscosity of the working fluid and its effect on the timescale of the system. The maximum pressure demonstrated in the actuator was 101 kPa. The frequency response of the system was reported for the oscillating beam as 0.01 Hz. To maintain similar timescales in a miniaturized soft robot, a less viscous working fluid would be necessary to maintain the coupling between the fluid and solid structures of the actuator. For a given scale, a less viscous material resulted in a slower normalized frequency. The mathematical approach in this paper provided full proportional control of the actuator’s position in time. No logic is possible, but the input pressure pulse could be used to reprogram the actuator’s response with multiple different modes of operation. The actuator was constructed from a polyurethane-based rubber. Since the control method was exclusively based on dynamics and introduced no additional structures to the robot, there were no negative effects on the robot’s mechanics. The beam was manufactured in a multi-step molding process which used a 3D printer to define the geometry of the first mold. This design is covered by US patent US10450051B2 (Gat et al., 2019).

In a work by Di Lallo et al. (2019), the damping design of soft actuators was investigated to provide control. The walls of soft pneumatic actuators were filled with silicone oil and 2 mm granular particles to tune the actuators’ damping responses. When supplied with a slow, ramped input pressure, both actuators with high viscosity (1,000 Pa · s) and low viscosity (0.5 Pa · s) silicone oil in their walls would inflate simultaneously. When supplied with a fast step-input pressure, the more damped actuator inflated significantly slower. The authors modeled the system by considering a finite element of the surface membrane in a soft actuator and solving for the geometric shear strain rate. The chamber filled with viscous fluid would then be subject to viscous shear stress proportional to both the fluid’s dynamic viscosity and the shear strain rate. The authors tested this concept by building extending, contracting, and bending actuators. In all cases, two DoFs could be controlled using a single external pressure connection. The actuators were all manufactured on the centimeter-scale, and the actuation method is believed to be difficult to scale to larger sizes due to the large volumes of compressible air that would arise. Conversely, miniature robots would require larger pressure ramp rates to effectively control the small volumes of air. The maximum pressure tested for any of the actuators was approximately 500 kPa. The slow pressure input resulted in inflation over a duration of 60  s, whereas the fast inflation took 5  s. This control method afforded binary control of the actuator states, as both the highly damped and the minimally damped actuators reached the same final inflation, but at different times. The control method exhibited no logic functions. The differently damped actuators allowed the system to be programmed between simultaneous and sequential inflation, but both actuators did reach the same state after the full duration of the pressure input. For the bending actuators, this was shown to be useful for achieving two different grasping configurations. The actuators were manufactured using EcoFlex 00–30 silicone (with a Young’s modulus of 125 kPa). This control method necessarily reduced the operating speed of the actuators to achieve simultaneous inflation, but the damping fluids did not otherwise seriously impact the actuators’ behavior. The molds for the actuators were manufactured using a 3D printer.

### 3.5 Controlling Soft Robots With Smart Fluids

Recently there has been great interest in exploring smart fluids for use in soft robots. These fluids have properties that can be directly modulated via external stimulus. We are primarily concerned with two similar, yet distinct, classes of smart fluids. Electrorheological (ER) fluids are comprised of micron-scale dielectric particles in a carrier liquid (Sadeghi et al., 2012). In the presence of an electric field, the micro-particles align causing the ER fluid’s viscosity and yield stress to increase, effectively solidifying the fluid. The effect is easily reversed by removing the electric field. Magnetorheological (MR) fluids are similar, but instead use micron-scale magnetic particles (Genc and Phule, 2002). The viscosity and yield stress of MR fluids increase in the presence of magnetic fields. In the absence of such fields ER fluids and MR fluids behave as Newtonian fluids. When a field is applied, they behave as Bingham plastics with a characteristic yield stress (Kenaley and Cutkosky, 1989; Helal et al., 2016; ; Jolly et al., 1996). When the yield stress increases, so does the pressure in the fluid flow. This can be used to control the pressure inside a soft robot. For both ER and MR fluids, the rheological effects scale with increasing electric and magnetic fields, respectively. Yield stresses for MR fluids range from 50 to 100 kPa versus a range of 3–5 kPa for ER fluids (Carlson et al., 1996). These properties can be tuned by varying the concentration of the active particles and changing the carrier fluid, balancing higher yield stress with increased density and viscosity (Wang et al., 2018). Various additives can also be added to improve the stability of the fluid and increase performance (Rendos et al., 2020a; Rendos et al., 2020b). Figure 3E depicts the working principle of a smart fluid as well as a soft robot controlled with an MR fluid.

ER fluids have been used to create valves suitable for controlling multi-actuator soft robots (Sadeghi et al., 2012). By placing planar copper electrodes on either side of a channel of flowing ER fluid, a strong electric field was delivered to solidify the fluid between each valve’s inlet and outlet. The fluid itself was composed of 50% dialectric particles with an average diameter of 3 µm in silicone oil (RheOil3.0, ERF Produktion Würzburg GmbH). The fluid had a base viscosity of 0.070 Pa · s. In the earliest use of this design, each actuator DoF required one valve that functioned as a two-way device. Pressure was applied and then removed using a single syringe pump with the valves dictating which actuators were controlled. Each valve had one pressure input, a wire for ground, and a wire for high voltage. The pressure inputs and ground wires could be shared among multiple valves. This was used to create a four actuator device for rotating a plate, a two actuator crawling robot, and a four actuator rolling robot. The valves were also demonstrated in this paper to be useful for stiffening a tendon actuated continuum robot arm. This valve design was refined by the authors in a later work (Tonazzini et al., 2016). Here, each actuator functioned by using one sub-valve to control inflow and another to control outflow of the ER fluid, thus forming a three-way valve system. Each valve pair had one pressure inlet, one pressure outlet, one ground wire, and two high voltage wires. Four fluidic actuators along with four inlet and four discharge valves were used to create a soft robot capable of axial extension and omnidirectional rotation in a three-dimensional workspace. The actuators shared a single inlet and a single outlet line to provide ER fluid flow through the robot. The channel between the electrodes within the valves had a width of 2.5 mm and a length of 15 mm. The channel valve gap varied from 0.25 to 1 mm, with smaller gaps able to withstand greater pressure while also increasing response time. With a field of 2 kV and a gap of 0.25  mm, as much as 1 MPa could be withstood by each valve. The ER effect itself had a response time of 14 ms, but the system took over 10  s for the maximum pressure to be reached. Since the ER effect scales with the applied electric field, this valve offered proportional control of the actuators. The valves were not used for any logic functions, but the input electrical signals could be controlled and changed in real time. In both papers, while the actuators were constructed of rubber (with a Young’s modulus on the order of 1–10 MPa (AZO Materials, 2021d)), the valves themselves were made of rigid materials, including aluminum (with a Young’s modulus of approximately 80 GPa (AZO Materials, 2021b)). A laser cutter was used to fabricate most of the valve components, except for the negative electrode which was machined from aluminum. A high voltage power supply was required offboard to control the electric field supplied to the valves.

A fully soft ER fluid valve has been presented more recently (Zatopa et al., 2018). The valve in this paper used eutectic gallium-indium (eGaIn) liquid metal to create soft electrodes. This paper also used RheOil3.0 as the ER fluid. The body of the valve itself was manufactured using a rubber-like 3D printed material. A stiffer material was used to reinforce the tube carrying ER fluid in the center of the valve where large expansions due to the build up of pressure would otherwise have occurred. The valve was demonstrated in an octopus-like robot with six coupled actuators which together shared one inlet and one outlet. A continuous flow of ER fluid at 140 kPa was provided at the inlet via a syringe pump, and the outlet drained to an open reservoir. The valve was located downstream of the actuators within the body of the robot. Applying the electric field to the valve at the shared outlet caused all the actuators to bend simultaneously. As such, the valve controlled one DoF. The valve required one ground wire and one high voltage wire. Since the fluid was continuously flowing, there were two pressure connections, one for the inlet and one for the outlet. The valve was approximately 4 cm long and 2 cm wide. The eGaIn electrode channels were 1 mm in diameter, and the ER fluid channel was 0.40 mm within the valve. With an applied voltage of 5 kV, the valve could hold pressures up to 264 kPa in its unstrained state. The system required approximately 10  s to reach the maximum pressure. The valve allowed for proportional control by controlling the voltage applied to the valve. The valve was not used to implement logic. The pressure in the actuators and thus their bending states could be controlled on-the-fly, but since they were coupled together the configuration of the robot was limited. The valve and actuators were printed using the FLX9750 rubber-like material on a Stratasys Objet350 Connex2 3D printer. The Young’s modulus was measured to be 0.95 MPa. In addition to the 3D printer, an oven was also used in the manufacturing of the valve. The valves required an offboard high voltage power amplifier and waveform generator to provide and control the strong electric fields.

MR fluids are gaining ground in the field due to their higher yield stresses when compared to ER fluids. Additionally, when actuated electromagnetically, MR fluids require far lower voltages than ER fluids, albeit at the expense of far larger power consumption. This particular problem was avoided in an article by Leps et al. (2020) where electropermanent magnets (EPMs) were used to generate magnetic fields. The large magnetic fields produced by these magnets were then used in valves that exploited the jamming behavior of an MR fluid to hold high pressures. Multiple MR fluids were manufactured and tested, but the most successful for the purpose of jamming effectively consisted of 34.5% iron particles, 64.2% mineral oil, and 1.5% oleic acid by volume. The iron particles themselves were a polydisperse distribution up to 45 µm in diameter. The valves, manufactured using a resin 3D printer, contained a constriction that allowed the MR fluid particles to form a stable blockage in the presence of a 220 mT field. Four of these valves were used to control the inflation of four 3D printed silicone actuators. Pressurized fluid was provided at a shared inlet. Depressurization was not demonstrated, but could also be accomplished through the same tube. With the magnets engaged, the valves prevented the actuators from pressurizing. The magnets were disengaged to allow fluid to fill the actuators. Each valve controlled one DoF, had one pressure connection, and had two wire connections. The valves were all connected to the same control board which allowed all of them to be controlled using just two wire connections overall. The valves also shared the pressure connection. The EPMs were 4 mm wide by 4 mm high by 6 mm long and could produce fields as high as 230 mT. The valve had an internal diameter of 1.93 mm. The valve could stop flows with pressures as high as 180 kPa and while jammed it was capable of holding pressures over 415 kPa. The time to stop flow after engaging the magnet was approximately 2  s. Due to the jamming behavior of the valves, the actuators could only be controlled between their on and off states with no intermediate configurations besides those afforded by the fill rate of the MR fluid into the actuators themselves. In this way, the valves demonstrated binary behavior, but no logic functions were shown. By using multiple valves together to control antagonistic actuators, the robot was demonstrated achieving multiple bending states. However, due to the limitation imposed by using a single bidirectional pressure input, the robot had to return to its initial state in order to reach alternate configurations. The actuators were manufactured using an SLA printer and a silicone urethane (Carbon3D, 2021) with a Young’s modulus of 3 MPa (Carbon3D, 2021). The valves were printed using a solid resin (Formlabs) with a Young’s modulus of 2.8 GPa (Formlabs, 2021). The EPMs were manufactured using multiple hard metals including mild steel (with a Young’s modulus of 205 GPa (AZO Materials, 2021a)). The valves were therefore significantly stiffer than the actuators. The end brackets for the EPMs were manufactured using a wire discharge machining process. As noted above, the valve constriction was fabricated using a 3D printer. Due to the onboard control board, only a low current connection was required to power the EPMs, so no specialized power supply was required.

An alternative approach to the use of MR fluids to control soft robots was presented by McDonald et al. (2020). In this paper, magnetic fields were applied to a continuously recirculating MR fluid to modulate the flow pressure and the bending of attached actuators. In this way the MR fluid provided both actuation and control. The MR fluid in this paper was comprised of 23% carbonyl iron particles, 75% deionized water, and 2% xanthan gum by volume. The iron particles themselves were 3–5 µm in diameter. The fluid’s pressure response was characterized using fields generated with an electromagnet. Rheometry was used to characterize the fluid to fit its behavior to the Bingham plastic model. Actuation of multiple soft actuators was demonstrated by varying the strength of the applied magnetic field without adjusting the flow rate. Several classes of soft actuators were demonstrated using permanent magnets to control the magnetic field including a 1-DoF actuator, a gripper with three coupled DoFs, and a device with two independent DoFs connected in parallel. The single actuator and gripper required one magnet to control actuation. The two independent actuators each required a magnet at the inlet and a magnet at the outlet. The paper also demonstrated a robot with five independent DoFs connected in series. Each DoF had an inlet and an outlet branching off from the shared recirculation channel. Placing a magnet at the actuator’s outlet engaged it. Placing a magnet at the actuator’s inlet bypassed it. To overcome the limitations of the series configuration, the space between the inlet and outlet on the primary recirculation channel had room for a magnet which could be placed to maintain equal resistance when the actuator was engaged. Each of these four actuator demonstrations, regardless of the number of DoFs, only required a single inlet and a single outlet. In each case, the channels were approximately 2 mm in both width and height. The permanent magnets were 12.5 mm in both thickness and diameter. The electromagnets used to quantify the pressure response consisted of 400 turns of 36 gauge copper wire wrapped into a 5 mm thick, 10 mm radius disc. The maximum pressure differential measured during the electromagnet tests was approximately 4 kPa, which was achieved with a rise time of 0.4  s. The magnetic field for this test was at most 20 mT, an order of magnitude less than the field produced by the EPMs used by Leps et al. (2020). This control method provided proportional control of the pressure in the actuators, and did not demonstrate any logic. The actuator states could be controlled in real time by adjusting the position of the magnetic fields, but this was accomplished manually. Although the magnetorheological effect was instantaneous with the application of the magnet, the actuators themselves took several seconds to inflate and deflate. The actuators were constructed from Ecoflex 00–30 (with a Young’s modulus of 69 kPa (Smooth-On, 2021c)) and Dragon Skin 20 (with a Young’s modulus of 338 kPa (Smooth-On, 2021b)). The magnets were comprised of nickel-plated neodymium (with a Young’s modulus of approximately 160 GPa (K&J Magnetics INC., 2021)). The magnets were therefore nearly 500,000 times stiffer than the robots, and would have imposed a significant restriction on the mechanics of the device if they had been fully integrated. The molds for all the actuators were laser cut from acrylic sheets and assembled with acrylic cement. The silicone was mixed using a planetary mixer and degassed in a vacuum chamber. An oven was used to cure the silicone. A spin coater was used to form a thin layer of silicone on the cured parts, which was subsequently used to bond the layers together. The MR fluid was mixed by hand.

## 4 Discussion

Each of the control methods discussed in this paper have their own advantages and use cases for which they are suitable. Traditional pneumatic components excel with regards to maximum pressure, bandwidth, and their ability to be reprogrammed. They additionally require no specific manufacturing considerations since they are readily available commercially. Microfluidic valves can control systems with many degrees of freedom, are highly scalable, and have a rich history of being used for logic both in soft robots and within adjacent fields of research. Macrofluidic pressure activated valves are excellent at reducing the number of external connections needed to control a robot and they have demonstrated a variety of complex logic behaviors. Control based on viscous effects is unsurpassed with regards to its minimal impact on robot mechanics and can be used to reprogram a robot via a single pressure input. Smart fluids can provide precise proportional responses and can control systems with many degrees of freedom. Table 2 provides a detailed summary of the Criteria for Comparison as they pertain to the different control methods.

**TABLE 2:** Summary of soft robot control methods and the criteria for comparison. For the number of external connections, the number listed is for pressure tubes, except for wires where noted. For scalability 5 stars represents dimensions below 1 mm, four stars is millimeter scale, three stars is up to approximately 1 cm in any dimension, two stars is up to 5 cm in any dimension, and one star is greater than 5 cm in any dimension. Bandwidths are all converted to frequencies in Hz for ease of comparison. Influence on robot mechanics is provided on a scale where 5 stars represents a method with little to no influence and one star represents a method which is substantially rigid. Manufacturing considerations are provided as a ranking of complexity for the end user. A 5 star method is an off-the-shelf component which requires no manufacturing, a three star requires only small equipment typically available in a soft robotics lab, and a one star method requires numerous manufacturing steps and specialized equipment such as that for lithography.

Control type	References	Number of DoFs	Number of external connections	Scalability	Maximum pressure [kPa]	Bandwidth [hz]	Binary vs. Proportional output	Use for logic	Ability to reprogram	Influence on robot mechanics	Manufacturing considerations
**Traditional**	Commercial Valves	1	varies	⋆ ⋆ ⋆ ⋆	34–827	1–200	B/P	No	Yes	⋆	⋆ ⋆ ⋆ ⋆⋆
**Pneumatic**	Moers et al. (2012)	1	1 (+2 wires)	⋆ ⋆⋆	600	1	B	No	Yes	⋆	⋆ ⋆⋆
**Components**	Marchese et al. (2011)	1	1 (+2 wires)	⋆ ⋆⋆	4.7	5	B	No	Yes	⋆	⋆ ⋆⋆
**Microfluidic**	Wehner et al. (2016)	2	2	⋆ ⋆ ⋆ ⋆⋆	50	0.092	B	Yes	No	⋆ ⋆⋆	⋆
**Valves**	Mahon et al. (2019)	2	3	⋆ ⋆ ⋆ ⋆	Vacuum	Not Stated	B	Yes	Yes	⋆ ⋆	⋆ ⋆⋆
	Bartlett et al. (2020)	16	5	⋆ ⋆ ⋆ ⋆⋆	90	Not Stated	B	Yes	Yes	⋆ ⋆⋆	⋆
**Pressure**	Rothemund et al. (2018)	Varies	Varies	⋆ ⋆	8	2	B	Yes	Yes	⋆ ⋆ ⋆ ⋆	⋆ ⋆⋆
**Activated**	Ikuta et al. (2012)	2	1	⋆ ⋆ ⋆ ⋆	400	0.025	P	No	Yes	⋆ ⋆	⋆ ⋆
**Valves**	Partridge and Conn (2020)	1	1	⋆	120	1.88	B	No	No	⋆	⋆ ⋆ ⋆ ⋆
	Miyaki and Tsukagoshi (2020)	3	1	⋆	80	0.6	B	Yes	No	⋆	⋆ ⋆ ⋆ ⋆
**Viscous**	Vasios et al. (2019)	4	1	⋆ ⋆ ⋆ ⋆⋆	102.7	0.4	P	No	Yes	⋆ ⋆ ⋆ ⋆⋆	⋆ ⋆⋆
**Effects**	Matia et al. (2017)	1	1	⋆ ⋆ ⋆ ⋆	101	0.01	P	No	Yes	⋆ ⋆ ⋆ ⋆⋆	⋆ ⋆⋆
	Di Lallo et al. (2019)	2	1	⋆ ⋆	500	0.017	B	No	Yes	⋆ ⋆ ⋆ ⋆⋆	⋆ ⋆⋆
**Smart**	Sadeghi et al. (2012)	1	1 (+3 wires)	⋆ ⋆⋆	1,000	0.1	P	No	Yes	⋆	⋆ ⋆
**Fluids**	Zatopa et al. (2018)	1	2 (+2 wires)	⋆ ⋆	264	0.1	P	No	No	⋆ ⋆ ⋆ ⋆	⋆ ⋆⋆
	Leps et al. (2020)	1	1 (+2 wires)	⋆ ⋆ ⋆ ⋆	415	0.5	B	No	Yes	⋆ ⋆	⋆ ⋆⋆
	McDonald et al. (2020)	5	2	⋆ ⋆⋆	4	2.5	P	No	Yes	⋆ ⋆	⋆ ⋆⋆

One major distinction among the various classes of controllers is the difference between those which are strictly mechanical and those that require electronics. Systems that operate through strictly mechanical means, such as the pressure activated valves in Section 3.3 among others, can be well suited to work in extreme environments such as search and rescue operations in radioactive settings. In such scenarios, electronics failures are more likely and could result in malfunctions. A strictly mechanical controller affords greater protection from environments hazardous to electronics.

Alternatively, electronically controlled robots, especially those using commercially available valves like those discussed in Section 3.1, are more similar to those in the field of rigid robotics. Electronically controlled systems are more compatible with traditional concepts of feedback and control, and a single robot can be applied to a greater range of tasks. This is often done at the expense of the robot’s flexibility and compliance, however.

No one valve or controller is suitable for every soft robot task. While some roboticists will value the high pressures and fast performances of commercially available valves, others will require solutions that are softer or smaller. The delicate mechanics of a soft robot allow some degree of control to be decentralized and integrated directly into the robot’s body and actuators. Such morphological computation allows soft robots to better replicate natural systems than rigid robots (Pfeifer et al., 2007; Zambrano et al., 2014). For this reason, recent works have seen an acceleration in the development of control methods which aim to minimize the impact on robot mechanics via materials-centric design. Smart fluids and control via viscous effects are still nascent techniques for controlling soft robots, although recent advances in the modeling of the fluid mechanics within soft robots may lead to more sophisticated control in the future (Breitman et al., 2020). While among the best techniques for preserving a robot’s compliance (with the exception of the field generating components in smart fluid control systems), their scalability has been underexplored. ER and MR fluids have been used in microfluidic applications with channels below 100 µm in diameter, which suggests they will be useful for miniaturized soft robots (Zhang et al., 2009; Whiteley et al., 2010). Additionally, progress towards the manufacturing of fully soft electrodes and coils may lead to entirely soft means of actuation (Lazarus et al., 2014; Do et al., 2018). Doing so may bridge the gap in the capabilities of electronically controlled and strictly mechanical systems. Future developments in soft robot control will no doubt borrow from diverse fields of research and synthesize new techniques and materials to continue pushing towards smaller, softer, more sophisticated systems.

A necessary step towards deployment of soft robots in novel applications is being able to embed onboard control. This is particularly relevant to soft fluidic robots which typically rely on bulky offboard systems to provide pressure. While such bulk is not a major factor for some soft robots, such as those used in manufacturing or static laboratory and clinical settings where offboard pressure controllers are not a major hindrance due to the robots’ stationary nature, onboard valves are an important component for autonomous, untethered robots, high-actuator density systems, and robots that operate in delicate environments where the overall system size is of primary concern. In this review, we gave an overview of the many techniques in use for integrating control onboard soft fluidic robots. We established a set of quantitative criteria for comparing the different control methods. In doing so we highlighted their individual strengths and weaknesses to serve as a tool for soft roboticists looking to incorporate control into their designs.
